# Rapid Detection of Volatile Organic Compounds by Switch–Scan Tuning of Vernier Quantum-Cascade Lasers

**DOI:** 10.1021/acs.analchem.2c04352

**Published:** 2023-01-26

**Authors:** Raphael Brechbühler, Miloš Selaković, Philipp Scheidegger, Herbert Looser, André Kupferschmid, Stéphane Blaser, Jérémy Butet, Lukas Emmenegger, Béla Tuzson

**Affiliations:** † Laboratory for Air Pollution/Environmental Technology, Empa, Überlandstrasse 129, 8600Dübendorf, Switzerland; ‡ Department of Chemistry and Applied Biosciences, ETH Zurich, Vladimir-Prelog-Weg 1−5/10, 8093Zurich, Switzerland; § Transport at Nanoscale Interfaces Laboratory, Empa, Überlandstrasse 129, 8600Dübendorf, Switzerland; ∥ Alpes Lasers SA, Avenue des Pâquiers 1, 2072St-Blaise, Switzerland

## Abstract

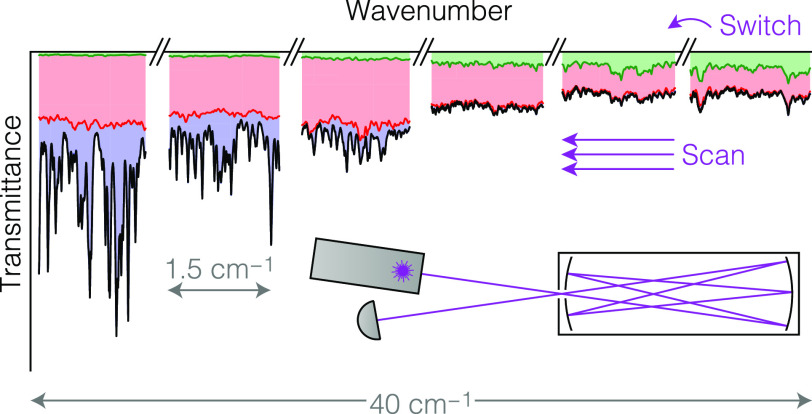

Volatile organic compounds (VOCs) exhibit typically broad and mutually overlapping ro-vibrational absorption fingerprints. This complexity has so far limited the applicability of laser-based spectroscopy for VOC measurements in complex gas matrices. Here, we exploit a Vernier-type quantum-cascade laser (QCL) as an electrically tunable multiwavelength source for selective and sensitive VOC analysis. This emerging class of lasers provides access to several spectral windows by discrete Vernier tuning (“switching”) and continuous coverage within these windows (“scanning”). We present a versatile driving technique that efficiently combines the two tuning mechanisms. Applied to our Vernier QCL, it enables the rapid acquisition (within 360 ms) of high-resolution spectra from six individual spectral windows, distributed over a wide range from 1063 to 1102 cm^–1^. Gaining access to the broad absorption envelopes of VOCs at multiple frequencies, along with their superimposed fine structure, which are especially pronounced at a reduced sample pressure, offers completely new opportunities in VOC analysis. The potential of this approach is assessed in a direct-laser-absorption setup with acetaldehyde, ethanol, and methanol as benchmark compounds with significant spectral overlaps. A measurement precision of 1–10 ppb is obtained after integration for 10 s at amount fractions below 10 ppm, and excellent linearity is found over at least 3 orders of magnitude. Combined with our dedicated spectral fitting algorithm, we demonstrate highly selective multicompound analyses with less than 3.5% relative expanded uncertainty, even in the presence of a 40× excess of an interfering compound with complete spectral overlap.

## Introduction

Small organic compounds with a high vapor pressure at room temperature are emitted by a wide range of biogenic and anthropogenic sources. The analysis of such volatile organic compounds (VOCs) is highly relevant for indoor and outdoor air monitoring, as they can have adverse environmental^1^ or health^2^ effects. Furthermore, they currently gain increasing interest in the analysis of exhaled breath for monitoring the metabolic state and for disease diagnosis.^3−5^ The standard methods to detect VOCs at trace levels are based on mass spectrometry with a wide range of ionization techniques (see, e.g., ref (6)). To promote the more widespread deployment of VOC monitoring, however, alternatives with lower complexity and reduced costs are required. In many situations, the analyzer should be compact, fast, and provide high selectivity and sensitivity.

Laser-absorption spectroscopy (LAS), which allows quantifying molecular species based on their unique absorption fingerprints, has the potential to satisfy these requirements. Of particular interest is the mid-infrared (mid-IR) spectral region because it contains the strongest ro-vibrational transitions. In this region, conventional distributed-feedback (DFB) quantum-cascade lasers (QCLs) are optimally suited light sources to probe the distinct and spectrally narrow absorption features of small, inorganic compounds in the gas phase.^7^ Indeed, trace-gas analyzers with high sensitivity^8^ and small footprint^9,10^ have been reported. The narrow laser tuning range of a few cm^–1^, however, significantly hinders extending laser-based mid-IR spectroscopy toward the detection of VOCs, which exhibit broad and congested absorption spectra,^11^ often with strong mutual overlap. Hence, laser sources are needed that maintain the advantageous properties known from DFB QCLs with an extended spectral coverage.

The currently available wide-tuning devices for the mid-IR region inherently exhibit various limitations. In particular, external-cavity (EC) QCLs,^12^ representing the most established solution with tuning ranges of up to several hundreds of cm^–1^, suffer from the hybrid integration of external optical and mechanically moving parts. This renders EC QCLs rather complex, costly, and susceptible to vibrations and temperature fluctuations. Continuous wavelength sweeping is prone to mode hops,^13^ and pulsed laser operation provides only limited spectral resolution. In addition, the repeatability and accuracy of the tuning are low (typically >0.1–0.5 cm^–1^).^14^ These characteristics render EC QCLs less suited for the specific detection of gas-phase VOCs because fine-structure details in their absorption spectra^15^ cannot be fully exploited. Methods based on supercontinuum sources have been reported,^16^ but to date they do not provide competitive sensitivities. Recent advances in mid-IR frequency combs promise fast and high-resolution measurements,^17^ yet they are demanding and costly.

In contrast, QCLs based on the Vernier effect (“Vernier QCLs”) are a promising alternative with monolithic integration, broad electric tunability, and the ability for rapid, high-resolution tuning. The cavity of these devices is defined by two distributed Bragg reflectors (DBR) that feature comb-like reflectivity spectra with slightly different peak spacing.^18^ Lasing requires a spectral overlap of two reflectivity peaks. This overlap is adjustable through Joule heating of the DBRs, which causes a refractive-index change that spectrally shifts the corresponding reflectivity comb. The general design of the Vernier laser offers two distinctive ways of wavelength tuning: (1) Discontinuous “switching” by displacing one peak with respect to the other until another pair of reflectivity peaks overlap, and (2) continuous “scanning” by simultaneously shifting the overlapping peaks.

The technology of Vernier QCLs has significantly matured over the last years. In particular, sophisticated reflector designs beyond the original sampled gratings^19,20^ have been proposed to widen the spectral coverage,^21^ tailor the spectral response,^22^ and enable cost-effective fabrication by deep-ultraviolet lithography.^23^ Additionally, laser driving has been simplified by separating the heating elements from the laser ridge to control Vernier tuning independent from laser-current injection.^23^ Despite these technical advances and the commercialization of Vernier QCLs (Alpes Lasers, Switzerland), the potential of this type of mid-IR laser for spectroscopic applications remained largely unexplored. Based on switching between a few discrete sampling points, measurements of solids^24^ and liquids^25^ have been reported. By additionally scanning the laser, small molecular species in the gas phase were analyzed. Bidaux et al. measured absorption lines of a single compound in multiple spectral windows as a proof of principle of Vernier lasers for spectroscopic applications.^23^ Diba et al. demonstrated simultaneous detection of multiple inorganic compounds with a low duty cycle,^26^ and Zifarelli et al. reported quartz-enhanced photoacoustic spectroscopy for nonsimultaneous analysis of inorganic trace gases.^27^ So far, organic compounds with strongly overlapping absorption fingerprints were beyond the reach of the measurements. The slow switching and idle times limited the acquisition speed and analytical performance, while the complexity of the driving hindered the widespread application of Verniers QCLs as attractive light sources.

In this work, we demonstrate selective and sensitive VOC detection using a Vernier-QCL-based absorption spectrometer. We address the existing limitations of the laser source and explore its potential by leveraging on a versatile driving scheme that efficiently combines high-resolution scanning with rapid switching. This switch–scan scheme was realized using custom-developed, low-noise driving electronics in combination with a data acquisition system based on a field-programmable gate array (FPGA). Our approach allows for the rapid acquisition of high-resolution spectral data in multiple spectral windows, mimicking several DFB QCLs integrated on a single chip. The method is assessed with acetaldehyde (AcH), ethanol (EtOH), and methanol (MeOH) as benchmark compounds. The compounds were analyzed at a reduced sample pressure (50 mbar), at which their strongly overlapping absorption spectra reveal significant fine-structure features. Although such distinctive signatures of VOCs can boost the performance of spectroscopic analyzers, they are not fully resolved in common spectral databases. Therefore, we generated custom reference spectra of the individual target compounds for use in our custom-developed global spectral fitting algorithm. To enhance the sensitivity and selectivity for complex gas matrices, this routine takes into account the combined spectral information obtained at multiple emission frequencies of the laser. Our approach successfully combines the stability, precision, and ease of use commonly known from single-mode DFB QCLs with a broad spectral coverage, allowing for an unambiguous and rapid measurement of VOCs even in complex gas matrices.

## Experimental Section

### Multi-Wavelength Vernier Laser

**Figure 1:**
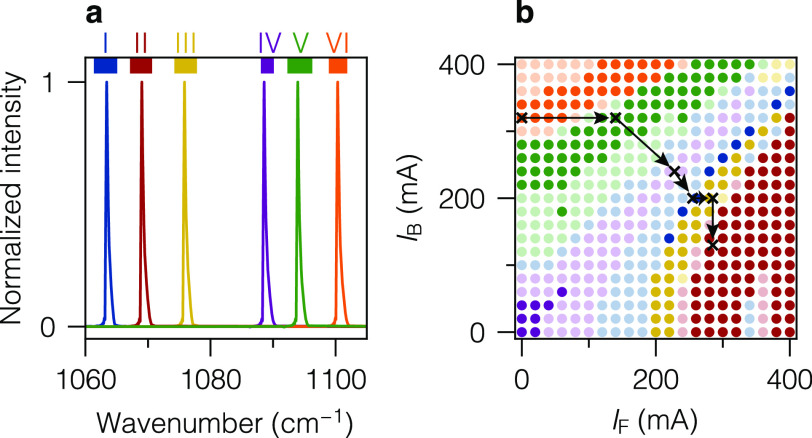
Characterization of the QC-XT laser, driven in the iCW regime and with CW heater currents (parameters provided in main text). (a) Laser emission at different heater configurations, measured with an FTIR spectrometer with 0.2 cm^–1^ resolution. Colored bars on top mark the six spectral windows (denoted as I–VI) accessible with the single-mode lasing configurations indicated in panel (b). (b) “Cluster map”. Dark (light) colored circles represent heater configurations resulting in single-mode (multimode) lasing output. Colors indicate the predominant cluster with the same color code as in panel (a). Black crosses and arrows denote the sequence of heater configurations selected for VOC measurements.

For this study we used a prototype eXtended-tuning QCL (QC-XT, Alpes Lasers) featuring buried semiconductor resistors as micro heaters integrated along the front and back DBRs. Their current supply is independent from the laser injection current (electronic circuit diagram in Figure S1), similar to the design reported in ref (23). Using the Vernier effect, the laser emission can be switched between individual lasing modes distributed in the range from 1063 to 1102 cm^–1^ (Figure 1a). In total, the device gives access to six different spectral windows through the specific combination of reflectivity peaks. We use the term “cluster” to denote these settings of lasing operation in different spectral windows. Thus, switching and scanning correspond to intercluster and intracluster tuning, respectively.

The laser was mounted in a modified water-cooled laboratory laser housing (LLH, Alpes Lasers) and operated at 0 °C in the intermittent-continuous-wave (iCW) regime^28^ with 150-μs-long current pulses (laser current, *I*_L_, of 700 mA) at a repetition rate of 3.33 kHz. By design of the QC-XT laser, the current pulse injected into the laser ridge red-shifts both the DBRs’ reflectivity peaks and the phase condition for lasing by the same amount, enabling smooth scans without separate phase tuning.^23^ The microheaters for the front DBR (hereinafter called “front heater”) and the back DBR (“back heater”) were driven individually. To find suitable combinations of front- and back-heater currents, *I*_F_ and *I*_B_, respectively, we have systematically analyzed the laser output at various heater configurations with a Fourier-transform infrared (FTIR) spectrometer (VERTEX 80, Bruker). The heater currents remained constant during the acquisition of each FTIR spectrum. Figure 1a shows representative emission spectra for configurations that result in single-mode lasing in different clusters. In the “cluster map” in Figure 1b, we classify the acquired FTIR spectra, sampled in heater-current steps of 20 mA, according to their emission wavelength with the same color code as in Figure 1a. The observed extended regions of the same color are expected from the general operation principle of the Vernier laser and motivate the term “Vernier clusters”. Dark-colored (light-colored) circles mark configurations with single-mode operation (side-mode contributions larger than 1 ‰ in intensity). From the FTIR spectra of all single-mode configurations, we extract the width of each accessible spectral window (colored bars on top of Figure 1a). At fixed operating parameters (especially laser temperature), there are gaps between the accessible spectral windows, which are, however, not detrimental for the purpose of this work.

### Laser Driving Scheme

**Figure 2:**
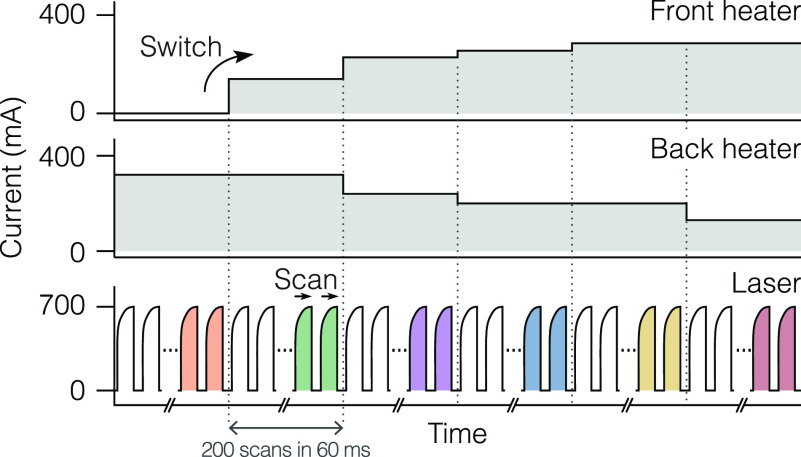
Driving scheme for QC-XT lasers. The schematic depicts one Vernier cycle (typically 360 ms duration). Switching between the Vernier clusters occurs at every heater-current step (top and middle panels). In parallel, the laser is repeatedly scanned within each spectral window by iCW driving with 150-μs-long current pulses (bottom panel). Typically, 200 scans were acquired per cluster and spectral data obtained from the last 100 pulses was averaged (visualized as colored laser pulses).

While Vernier QCLs have previously been explored for LAS,^23−26^ rapid switching between multiple clusters combined with high-resolution spectral scanning within the individual clusters has not yet been demonstrated. Switching after every single scan leads to significant idle times because of the slow thermal response upon heater-current changes (duty cycle of ∼1%^29^). To enable measurements with a high signal-to-noise ratio, we designed a tailored driving scheme with significantly higher throughput. Within one measurement cycle of this scheme, the current levels of the front and back heaters are stepwise modified to switch between the Vernier clusters (top and middle rows in Figure 2). In parallel, we acquire many spectral scans by driving the laser in the iCW regime (bottom row in Figure 2) with a much faster repetition rate than the switching rate. The scans within a given cluster are nominally identical and can readily be averaged. Unless stated otherwise, we included all six Vernier clusters and stepped between them every 60 ms (∼16.7 Hz) while scanning with a pulse repetition rate of 3.33 kHz. This yields 200 high-resolution spectral scans per Vernier cluster, from which the first half was typically omitted in the averaging process to minimize spectral distortions due to transient thermal effects. The resulting duty cycle is 25%.

In previous work on the QC-XT laser, much longer waiting periods of up to 15 s were required after switching, caused by the stabilization time of the temperature controller of the laser.^25^ We efficiently overcome this problem in our scheme by an optimum choice of cycle parameters. First, fast (>15 Hz) switching between the heater configurations ensures that the temperature controller predominantly acts on the cycle-averaged temperature. Second, we used the cluster map (Figure 1b) to select configurations that provide access to the six Vernier clusters at comparable total heating power, that is, with similar *I*_F_^2^ + *I*_B_^2^. This minimizes the variations in the heat load throughout the cycle. For optimum performance, the selected heater levels were then fine-tuned to maximize the laser intensity and optimize the side-mode suppression. In Figure 1b, the black crosses and arrows between them represent the final sequence of heater-current levels used for the Vernier cycle, applied throughout this paper, to subsequently access the spectral windows in the order VI, V, IV, I, III, and II. For window IV, single-mode operation was achieved in a narrow range of heater-current levels along the diagonal in Figure 1b, which is not resolved in the cluster map due to the coarseness of the step size.

### Driving and Data-Acquisition Electronics

Three current driver modules were connected to the laser device for independent laser-current injection and operation of the two individual micro heaters (schematic and details in Figure S1). These custom-developed driver modules are based on an earlier design,^30^ but with Vernier-specific adaptations to enable rapid switching between different output currents and provide full galvanic isolation. The latter is necessary because the QC-XT laser device must be operated with a floating ground. In each module, the current switching was implemented with an analog multiplexer, which forward one out of five control signals (corresponding to four programmable current levels and no current) to an integrated current driver. For the laser driver, the current ramp was further shaped with two programmable resistor–capacitor circuits to reduce the tuning rate at the beginning of the iCW pulse.^28^

The switching of the individual driver modules was precisely triggered and synchronized by controlling the multiplexers with an FPGA on a programmable board (Alpha250, Koheron). The same board processed the signal from the infrared detector after digitization with an integrated analog-to-digital converter (ADC, 250 MSa/s, 14 bit). The scans within each spectral window were averaged onboard the FPGA (with the option to discard the first scans after switching). The data were then transferred into the DDR-RAM of the processing unit, buffered, and sent via a TCP/IP interface to an external computer after the completion of a full Vernier cycle for spectral analysis.

### Spectrometer Design

**Figure 3:**
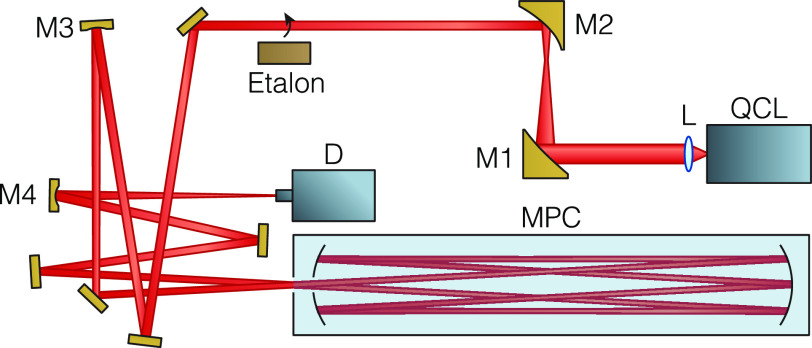
Schematic optical layout of the spectrometer. The optical elements used to shape and steer the laser beam are indicated as follows: L denotes the aspheric lens with focal length 12.7 mm; M1 and M2 are OAP mirrors with reflective focal lengths of 3 and 1 in., respectively; M3 and M4 are concave mirrors with focal lengths of 500 and 50 mm, respectively. QCL and D represent the Vernier laser and the infrared detector, respectively.

Figure 3 depicts a schematic of the spectroscopic setup. The laser output was collimated with an aspheric ZnSe lens (AL72512-E3, Thorlabs), and then its beam size was reduced by 3× using a pair of off-axis parabolic (OAP) mirrors (MPD139-M01 and MPD119-M01, Thorlabs). After passing through additional beam steering and shaping elements, the beam was coupled into an astigmatic Herriott multipass cell (AMAC-76, Aerodyne Research, Inc.), providing an optical path length of 76 m. The output beam after the multipass cell (MPC) was focused onto a fast, thermoelectrically cooled infrared detector with optical immersion lens (PVMI-4TE-8-0.7×0.7-TO8-wBaF2-70, Vigo System S.A.). The detector signal was internally preamplified (PIP-DC-200M-F4, Vigo System S.A.) and low-pass filtered (frequency cutoff 78 MHz, ZX75LP-70-S+, Mini-Circuits). All optical components were mounted on an optical breadboard, and the system was enclosed with a thermally insulated box.

### Gas-Delivery System

For the validation of the spectrometer, AcH, EtOH, and MeOH served as benchmark VOCs. Certified calibration gases of these compounds diluted in dry N_2_ were obtained in pressurized gas cylinders (PanGas) at amount fractions of 199.4, 200.0, and 200.3 μmol/mol (or using the dimensionless unit part per million, ppm) with 5, 2, and 5% relative expanded uncertainty (*k* = 2), respectively. Additional gas cylinders of CO_2_ (grade 4.5, PanGas) and N_2_ (grade 5.0, PanGas) were used for absolute-frequency calibration and as dilution gas, respectively. Furthermore, CO_2_-free air was used for purging of the gas-delivery system (CO2-PG14-2 purge-gas generator, Altec AIR). The sampling line consisted of a multiport selector (EUTB-2SD6MWE, VICI AG International) and mass-flow controllers (GSC series, Vögtlin Instruments GmbH; MFC 2000 series, Axetris) for dynamic dilution and delivery of the gases at a constant flow rate through the MPC. A precision needle valve mounted downstream of the MPC toward the pump (PM30611-920.18, KNF) provided control over the cell pressure, which was monitored with an absolute pressure transducer (MKS Baratron 722B, MKS Instruments, Inc.). The temperature of the MPC housing, a proxy for the sample temperature inside the cell, was probed with a 10 kΩ thermistor.

### Spectral Analysis

We developed a LabVIEW-based software suite for hardware control and data processing. The conversion of the digitized raw detector signal (cf. typical signals in Figure S2) to a transmission spectrum has been described previously.^15^ In brief, first the detector offset, recorded before the start of the laser emission, was subtracted from the signal. Then the signal was normalized by the transmission spectrum of the MPC without absorbing compounds. A relative-frequency scale was obtained from a transmission spectrum of a 2 in. long Ge etalon placed into the beam path (cf. Figure 3). The absolute-frequency scale was calibrated by referencing a transmission spectrum of CO_2_ diluted in N_2_ to spectral data from the HITRAN database.^31^

Custom reference spectra were generated for each individual compound, because neither spectra at sufficiently high resolution nor line-by-line absorption parameters (reported for MeOH in a part of the examined range) were available in common spectral databases. Thereby, we followed the nomenclature of the PNNL reference spectral database,^11^ i.e., the generated reference spectra correspond to absorbance for a sample amount fraction (*y*_ref_) of 1 ppm over an optical path length (*l*_ref_) of 1 m at a pressure (*p*_ref_) of 1 atm and a temperature of 296 K (*T*_ref_). Therefore, our transmission data of compound *i* at wavenumber ν̃, , was converted to absorbance  and normalized as
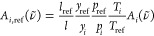
1where *l* is the optical path length of the MPC, *y*_*i*_ is the amount fraction of compound *i*, and *p*_*i*_ and *T*_*i*_ denote the measured sample pressure and temperature, respectively.

**Figure 4:**
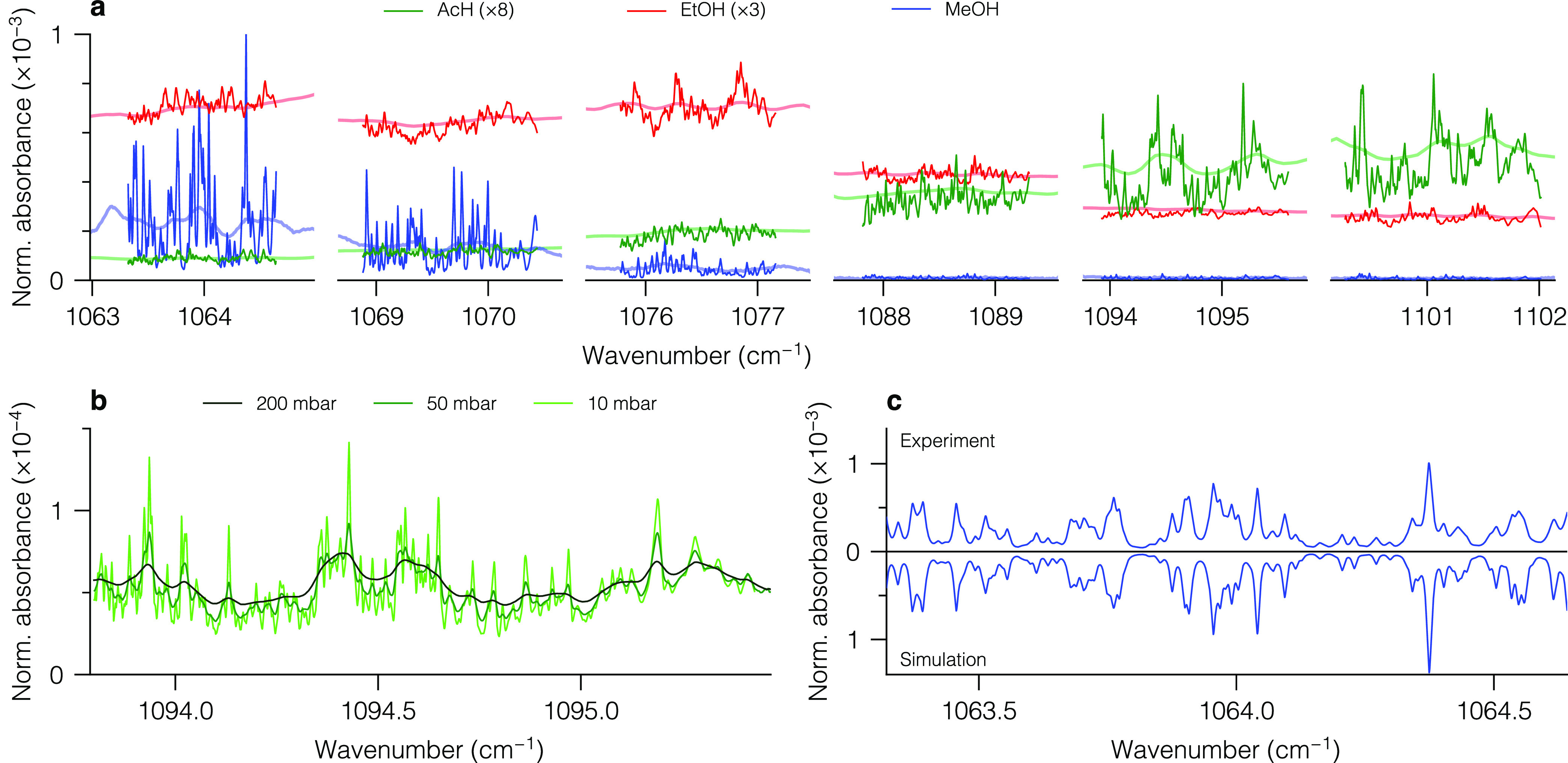
Absorption spectra of individual benchmark compounds. (a) Experimental data for AcH (green), EtOH (red), and MeOH (blue), along with available FTIR data (light-colored lines). The high-resolution spectra obtained with the QC-XT laser feature a large amount of superimposed fine structure. Applied scaling factors for EtOH and AcH are mentioned in the legend. (b) Experimental absorption spectra for AcH in spectral window V at different sample pressures. (c) Experimental (top) and simulated (bottom) absorption spectra for MeOH in spectral window I. Line-by-line parameters for the simulation in panel (c) and FTIR data in panel (a) were obtained from ref (31). All spectra are normalized as described in the main text.

Next, the generated reference spectra (examples depicted in Figure 4a) were used to quantify the amount fractions of compounds in gas mixtures by fitting. In the first step, the spectra obtained from the individual spectral windows were evaluated using the framework described in ref (15). Namely, the measured spectrum from each window was separately fitted with a modeled multicompound transmittance, calculated from the reference absorption spectra rescaled to the experimental conditions and weighted by the respective amount fractions as fitting parameters. Minor changes in the laser intensity and potential frequency drifts were taken into account in the model as a polynomial function and an absolute-frequency offset, respectively, as additional fitting parameters. The fitting parameters for each spectral window were optimized through a nonlinear least-squares algorithm based on the Levenberg–Marquardt method, to minimize the sum of the squared errors between the experimental data points and the modeled spectrum. Such single-window fits can only provide reliable estimates for compounds that feature a significantly strong and unique absorption fingerprint in the covered spectral range. Therefore, a robust estimate was obtained in a second step by fitting the entire spectral information from multiple windows of the Vernier cycle globally at once (example in Figure S3). For this “global fit”, the laser-intensity corrections and the mean results of the retrieved single-window amount fractions were applied as starting parameters. The previously determined absolute-frequency offsets were not further modified.

### Validation of the Method

The performance of the laser spectrometer was assessed for precision, linearity, and selectivity. The measurements were performed at a sample pressure of around 50 mbar.

*Precision*: The individual VOCs diluted in N_2_ at amount fractions of around 10 ppm were measured for 3 h at a constant total flow rate of 250 mL/min. The last hour of the measurement data was taken to determine the precision of the system.

*Linearity*: A series of binary gas mixtures of a VOC and N_2_ was prepared by dynamic dilution at amount fractions ranging from 150 ppb to 200 ppm. Each step was analyzed for 10 min at a constant total flow rate of 250 mL/min and repeated in triplicate. For each compound, the sequence was realized in one run and in random order.

*Selectivity*: A series of multicompound gas mixtures of the VOCs and N_2_ was prepared to investigate the selectivity of the method. The trinary mixtures consisted of two VOCs and N_2_. In those mixtures, one VOC was kept at a constant amount fraction of ∼4 ppm, while the other compound was varied from ∼4 to ∼160 ppm. In this way, mixtures of AcH:MeOH, AcH:EtOH, and EtOH:MeOH with amount ratios between 1:40 and 40:1 were realized. Each gas mixture was measured for 10 min at a constant total flow rate of 250 mL/min. The sequence of measurements was realized in one run and in random order for every pair of VOCs.

## Results and Discussion

### Absorption Spectra

The QC-XT-based spectrometer was used to measure absorption spectra of the individual benchmark VOCs (amount fractions of ∼200 ppm, sample pressure of 50 mbar). Each panel in Figure 4a represents the averaged scans over ∼1.5 cm^–1^ in one of the Vernier clusters. The span between 1063 and 1102 cm^–1^, corresponding to a relative wavelength range of 3.6%, covers regions of strong absorption for all studied compounds. For visual comparison, spectra taken from the HITRAN database^31^ are overlaid as light-colored lines. These FTIR spectra have a resolution of 0.015 cm^–1^ for MeOH^32^ and 0.112 cm^–1^ for AcH and EtOH,^11^ respectively, and were acquired at ambient conditions, i.e., at room temperature and atmospheric pressure. While the broad absorption envelopes of the two data sets qualitatively match each other, our spectroscopic data reveals a large amount of significant fine structure due to the higher spectral resolution and the reduced sample pressure. The benefits of prominent fine-structure details for VOC detection are twofold: (i) the additional spectral contrast between different gases beyond their broad absorption envelopes facilitates selective multicompound measurements, and (ii) the absorption can more easily be distinguished from potential fluctuations of the laser intensity that appear as slow baseline variation, thus enhancing the measurement precision. Figure 4b shows the effect of pressure broadening on the fine structure for AcH. Clearly, the features are most pronounced at low gas pressures. Similar trends were also found for the other benchmark compounds. The pressure dependence of the spectra highlights that fitting based on reference data requires a matched sample pressure. The choice of an optimum pressure is, however, inherently a trade-off because the number density of absorbing molecules, and thus, the signal-to-noise ratio of the measurement, increases with sample pressure.

A direct comparison of our measurements with reported high-resolution spectral data is only possible for MeOH, for which the HITRAN database^31^ provides line-by-line spectroscopic parameters for a part of our covered range. Figure 4c shows our experimental data for MeOH (top) along with the simulated spectrum using line-by-line parameters (bottom). The good match between the spectra supports the quality of our method and the data treatment. The minor broadening effect toward higher wavenumbers in our spectrum is caused by the low bandwidth of the detector’s preamplifier, which was chosen to minimize high-frequency noise. However, as this broadening is systematic, it will not affect the concentration-retrieval algorithm based on reference spectra generated with the same instrument.

### Precision

**Figure 5:**
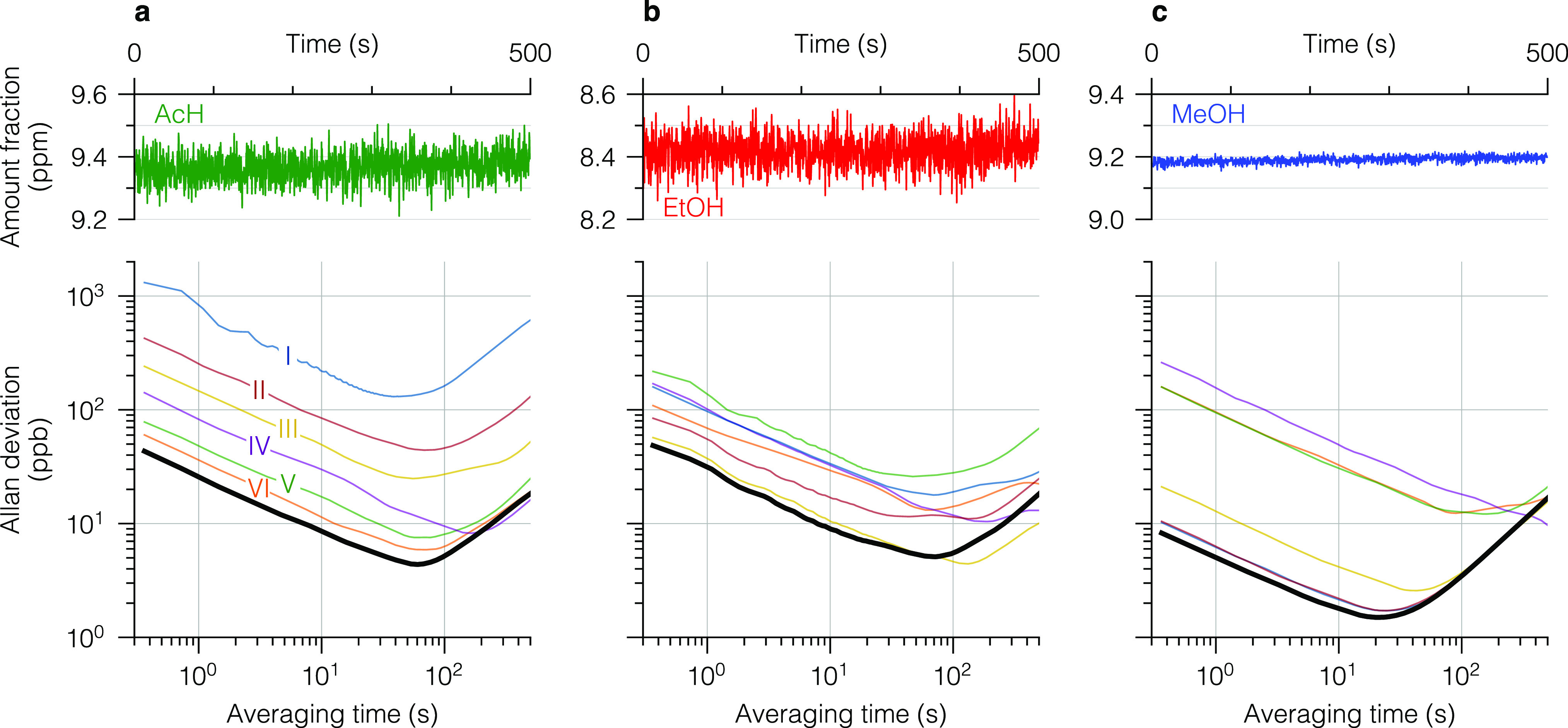
Measurement precision for individual benchmark compounds (a) AcH, (b) EtOH, and (c) MeOH. (Top) Time series with time resolution of 360 ms of retrieved amount fractions using global fitting. (Bottom) Allan deviation of fitted amount fractions as a function of averaging time at amount fractions of around 10 ppm. Black (colored) lines correspond to global (single-window) fitting. The single-window fits are labeled with the corresponding spectral windows and have the same color code as in Figure 1a.

Figure 5 summarizes the measurement precision of our spectrometer for individual compounds. From the globally fitted amount fractions with 360 ms time resolution (top panels in Figure 5), we calculated the Allan deviation^33^ (black lines in bottom panels). The minimum of each curve represents the best precision (1 σ) and the corresponding integration time, i.e., 4.5 ppb at 60 s, 5 ppb at 70 s, and 1.5 ppb at 20 s for AcH, EtOH, and MeOH, respectively. For each compound, we also depict the Allan plots for the six single-window fits as light-colored lines. In general, the global fit outperforms the individual single-window fits because it takes advantage of the full spectral information obtained from the entire Vernier cycle. The single-window fits, in contrast, only rely on a subset of this data. As expected, the best-performing fits are obtained in the spectral ranges with the strongest absorption and the most prominent fine structure, which depend on the measured compound (cf. Figure 4a). This implies that the driving strategy can be further tailored for specific compounds and applications.

### Linearity

**Figure 6:**
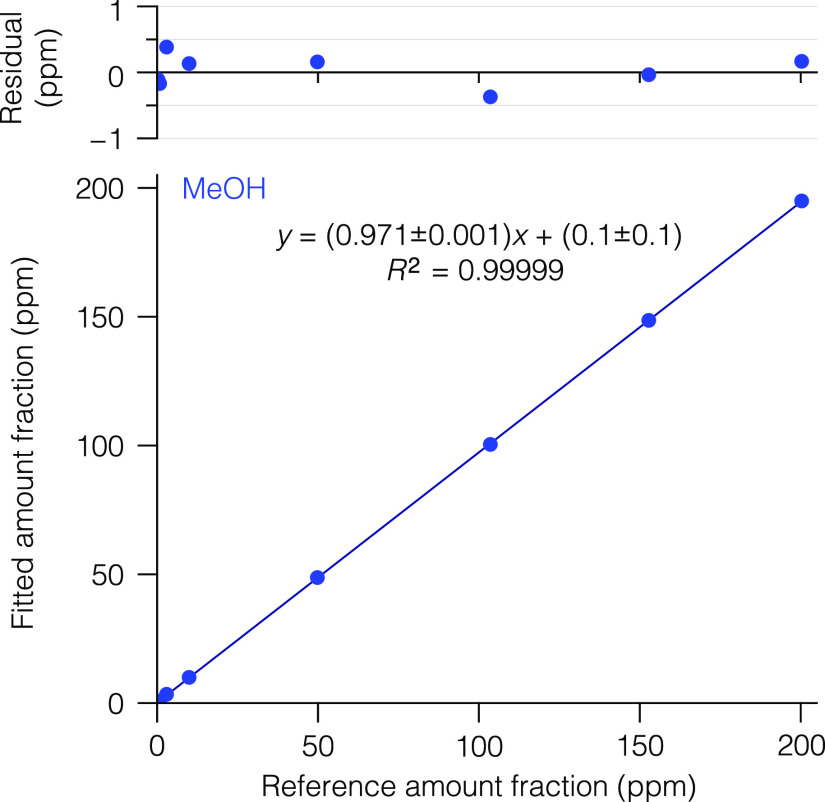
Linearity plot of MeOH (bottom) along with the corresponding residuals (top). The solid line shows a least-squares linear regression. The error bars (two-sigma deviation) are smaller than the marker size.

The linearity of the spectrometer was assessed for individual VOCs dynamically diluted to different amount fractions. Figure 6 shows the measured amount fraction of MeOH retrieved by global fitting, using the certified calibration gases at around 200 ppm as reference spectra (cf. Figure 4), as a function of the reference value. The calibration graphs for the other benchmark compounds (Figure S4) showed similar behavior. Several statistics indicate very good linearity of the instrumental response over the examined range of more than 3 orders of magnitude, in particular, a high value of Pearson’s coefficient, a random distribution of the residuals (except AcH with a noticeable trend) without bias, and a low standard deviation of the individual measurements. This allows for a single-point calibration, and thus, fitting based on a single reference spectrum is sufficient for the entire examined range. For all three compounds, we observed a deviation of the slopes of the calibration curves from unity of about 3%. We attribute this discrepancy to complex adsorption dynamics and memory effects in our gas-delivery system, which were not fully reproducible over the course of multiple days. In general, we observed long equilibration times for the investigated VOCs with complex transients that depend on the composition of the gas mixture, amount fraction(s), flow rate, and pathway through the gas-delivery system. A careful design of the inlet system, depending on the targeted application and compounds, could further improve the accuracy of the measurements.

### Selectivity

**Figure 7:**
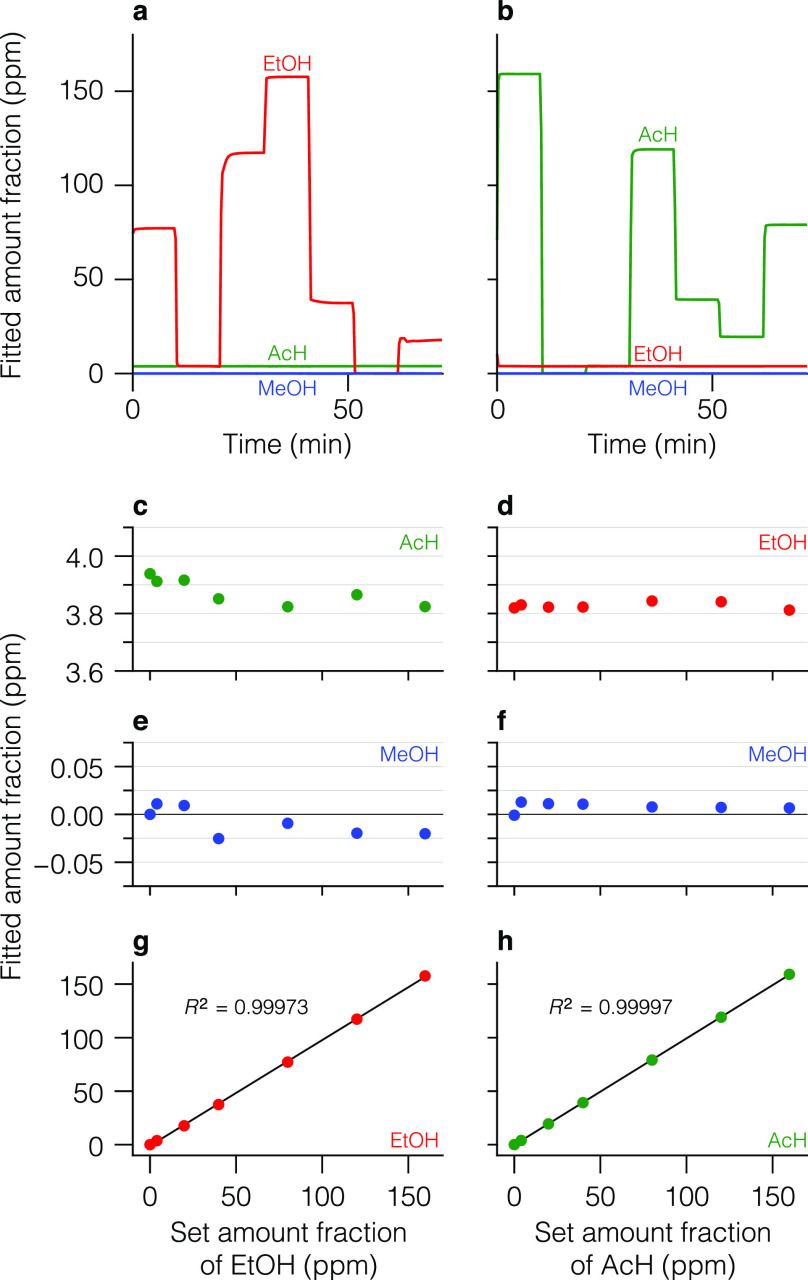
Selectivity study on the example of AcH–EtOH mixtures at amount ratios between 1:40 and 40:1. (a) Time series of fitted amount fractions of the three benchmark compounds when AcH is kept constant at around 4 ppm while the amount fraction of EtOH is varied between 0 and 160 ppm, and MeOH is absent. (c, e, g) Fitted amount fractions (colored dots) of the three compounds, averaged over the last 3 min of each step in panel (a) and ordered as a function of the set amount fraction of EtOH. (b) Same as (a) but with EtOH and AcH as the constant and the varied compound, respectively. (d, f, h) Same as (c), (e), and (g), but extracted from panel (b) and ordered as a function of the set amount fraction of AcH. The solid lines in panels (g) and (h) represent the fitted linear regression.

As an ultimate challenge, we analyzed gas matrices consisting of VOCs with spectrally overlapping absorption features. Figure 7 shows a typical selectivity experiment on AcH–EtOH mixtures with amount ratios between 1:40 and 40:1 (similar plots for other combinations in Figures S5 and S6). For the data analysis, all three benchmark compounds were included in the fit despite the absence of one compound (e.g., MeOH in Figure 7e,f). The time series (Figure 7a,b) qualitatively reveal an excellent selectivity of the method.

To analyze the cross talk among the compounds, we plot the fitted amount fraction of the constant compound as a function of the set amount fraction of the varied (interfering) compound. AcH shows a slight cross talk in the presence of an up to 40× excess of EtOH in Figure 7c (of MeOH in Figure S5d), which affects the fitted amount fraction by less than 120 ppb (60 ppb). This corresponds to a relative uncertainty of 2.9% (1.5%), defined as the maximum deviation of the fitted amount fraction introduced by the interference. For all other cases, no systematic cross talk is observed, yielding a relative expanded uncertainty (*k* = 2) of 0.7% for EtOH in the presence of AcH (Figure 7d) and below 3.5% in general (Figures S5c and S6c,d). Here, A- and B-type uncertainty are defined as the standard deviation of the data points and the difference between mean and reference value, respectively. As expected, we found that no single spectral window could provide a comparably good selectivity of the method. This is likely a consequence of the absence of a common spectral window in which the compounds simultaneously exhibit well-defined features and strong absorption. This again highlights the benefit of the wide tuning capabilities of the QC-XT laser. Finally, the excellent linearity of the method is preserved in complex matrices (cf., panels g and h in Figures 7, S5, and S6).

## Conclusions

We have developed and validated a high-performance laser spectrometer for VOC detection based on a Vernier-type QCL. A versatile switch–scan laser driving scheme is proposed that successfully combines high-resolution spectral scanning, as known from DFB QCLs, with rapid switching between different spectral windows. The augmented spectral coverage by switching was obtained while maintaining state-of-the-art sensitivity of laser-based mid-infrared spectroscopy.

Our assessment shows that the approach is well-suited for the selective analysis of complex mixtures of VOCs, despite their broad and spectrally overlapping fingerprints. This becomes possible by fully exploiting the often neglected fine structures in the absorption spectra, which gain prominence especially at reduced sample pressure. Probed over the wide range of the laser (spanning around 40 cm^–1^), these features significantly contribute to the demonstrated excellent selectivity of the method.

Because the spectral information from the entire tuning range is obtained on subsecond time scales, our approach will also be beneficial for multicompound analyses in dynamic systems, including online monitoring, rapid quality control, and inspection. Here, laser spectroscopy based on Vernier QCLs could become highly complementary to established VOC-detection methods thanks to its quantitative and fast response, comparably low instrument and maintenance cost, and compactness. More generally, the presented approach offers new avenues to further expand the field of applications for laser-based absorption spectroscopy for a large variety of environmental, industrial, and medical applications.

## Supporting Information Available

Additional details on the electronic setup, representative raw spectral data, and results of multispectral fitting and additional linearity and selectivity data supporting the validation of the method (PDF)
