# Supplementary material for: Rapid Detection of Volatile Organic Compounds by Switch–Scan Tuning of Vernier Quantum-Cascade Lasers
Source: Anal Chem. 2023 Jan 26;95(5):2857–64. doi: 10.1021/acs.analchem.2c04352 (PMC9909733; doi:10.1021/acs.analchem.2c04352)
Supplement: Supplementary file 1 — ac2c04352_si_001.pdf [file ac2c04352_si_001.pdf]

Supporting Information
Rapid detection of volatile organic compounds by switch-scan tuning of Vernier quantum-cascade lasers

Raphael Brechbühler,[†] Miloš Selaković,[†,‡] Philipp Scheidegger,[†] Herbert Looser,[†] André Kupferschmid,[¶] Stéphane Blaser,[§] Jérémy Butet,[§] Lukas Emmenegger,[†] and Béla Tuzson*,[†]

†*Laboratory for Air Pollution / Environmental Technology, Empa, Überlandstrasse 129, 8600 Dübendorf, Switzerland*

‡*Department of Chemistry and Applied Biosciences, ETH Zurich, Vladimir-Prelog-Weg 1–5/10, 8093 Zurich, Switzerland*

¶*Transport at Nanoscale Interfaces Laboratory, Empa, Überlandstrasse 129, 8600 Dübendorf, Switzerland*

§*Alpes Lasers SA, Avenue des Pâquiers 1, 2072 St-Blaise, Switzerland*

E-mail: bela.tuzson@empa.ch

Phone: +41 58 765 4642

Table of Content

Figure S1	Schematic of the electronic setup	S3
Figure S2	Representative digitized raw detector signals	S4
Figure S3	Multi-spectral fitting using custom-generated reference spectra	S5
Figure S4	Linearity plots for AcH and EtOH	S6
Figure S5	Selectivity study for AcH–MeOH mixtures	S7
Figure S6	Selectivity study for EtOH–MeOH mixtures	S8
Figure S7	Drift correction	S9

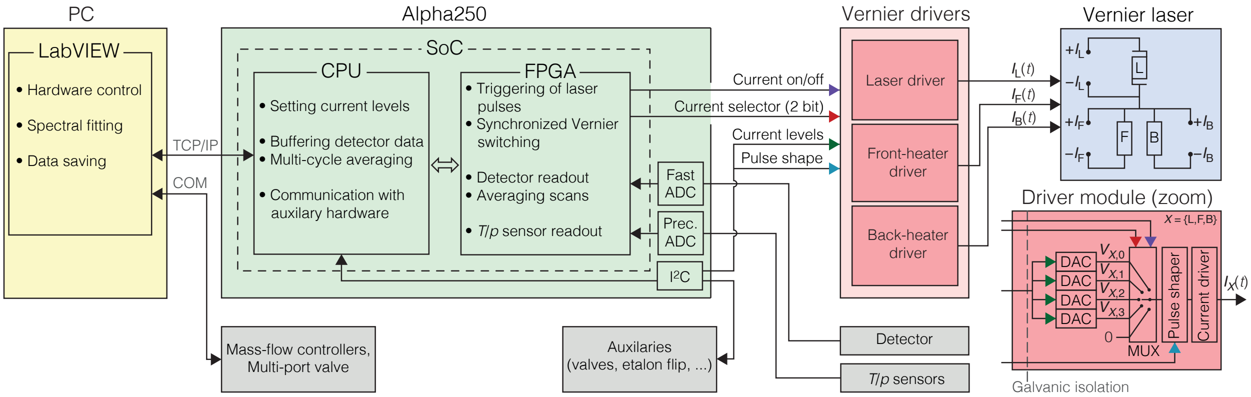

Figure S1: Schematic of the electronic setup. A simplified electronic circuit diagram of the QC-XT laser is depicted in the blue rectangle. The laser is driven with three separate currents, $I_\mathrm{L}(t)$, $I_\mathrm{F}(t)$, and $I_\mathrm{B}(t)$, through the active area, the front heater, and the back heater, respectively. The currents are supplied by three custom-developed driver modules (red rectangles). In each module (inset on bottom right), one out of five control voltages (the programmable levels $V_{X,0-3}$ and 0) is forwarded to the output line of an analog multiplexer ("MUX") to generate a control signals with the desired amplitude and pulse length. Next, the shape of the control signal is adjusted with two programmable resistor–capacitor circuits to reduce the tuning rate at the beginning of the iCW pulse ("pulse shaper"). This signal is then fed into the driver stage ("current driver") that generates the output current $I_X(t)$. The driver modules feature a full galvanic isolation to enable laser operation with floating ground. The switching of the driver modules was precisely triggered and synchronized using a field-programmable gate array (FPGA) on a programmable board (Alpha 250, Koheron), depicted in green. The board also digitized the signal from the infrared detector and from the pressure and temperature sensors through analog-to-digital converters, "fast ADC" (250 MSa/s, 14 bit) and "precision ADC" (1 Sa/s, 24 bit), respectively. For each spectral window, the scans were averaged onboard the FPGA, and then transferred to the DDR-RAM of the processing unit ("CPU") on the system on chip ("SoC"), buffered, and sent to an external computer (yellow rectangle) after the completion of a full Vernier cycle *via* a TCP/IP interface for spectral analysis.

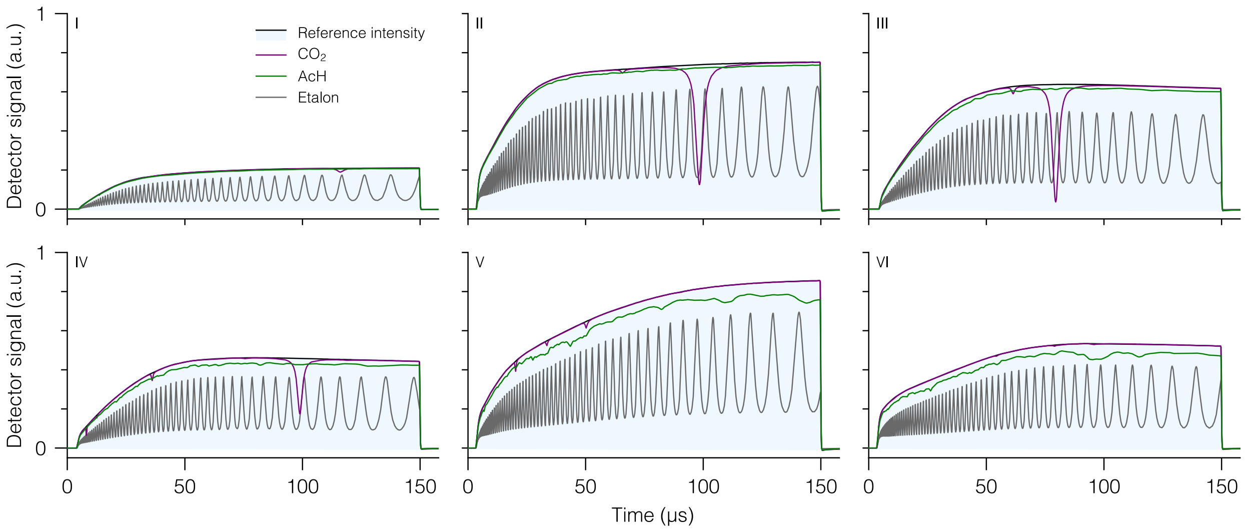

Figure S2: Representative digitized raw detector signals in the six spectral windows (labeled with Roman numerals), which were used to generate reference spectra as described in the main text. The transmission signal of the MPC without absorbing compound (black lines) serves as reference signal for laser-intensity normalization. The gray lines denote transmission spectra of a 2-in-long Ge etalon flipped into the beam path. The distance between two subsequent maxima in such an etalon trace, the free-spectral range (FSR), corresponds to a frequency tuning by $0.024379\,\mathrm{cm}^{-1}$ and is used to establish a relative frequency scale for each spectral window. The purple and green lines correspond to transmission signals in the presence of CO_2 and AcH, respectively. The distinct absorption features of CO_2 are used for absolute-frequency calibration within each spectral window.

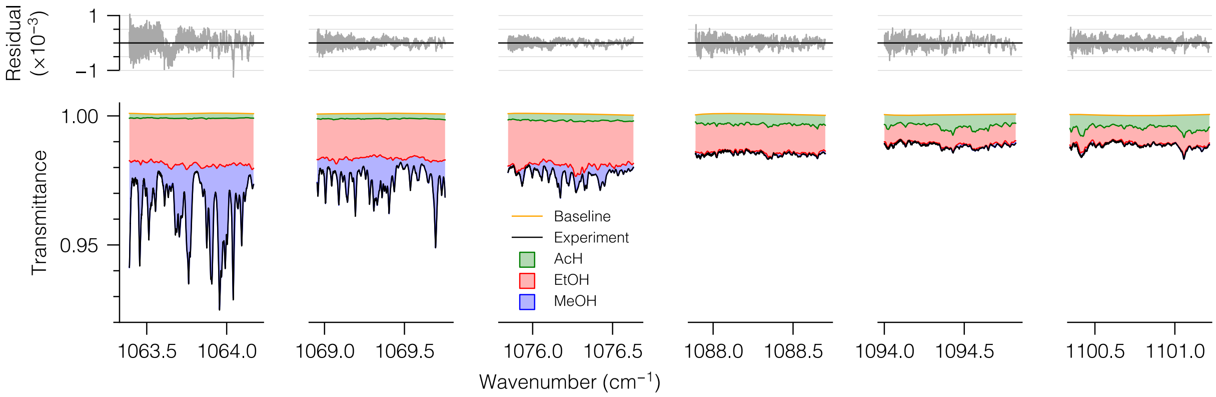

Figure S3: Multi-spectral fitting using custom-generated reference spectra of a gas mixture consisting of AcH, EtOH, and MeOH in N_2 at a sample pressure of around 50 mbar. The experimental data (black lines) is averaged over 50 subsequent Vernier cycles, corresponding to an integration time of 18 s. The colored areas represent globally fitted contributions by the individual compounds to the modeled transmission, corresponding to 9.66, 9.23, and 9.16 ppm of AcH, EtOH, and MeOH, respectively. The orange lines represent the determined baselines in the individual spectral windows. The top panels show the residuals between the experimental data and the globally fitted model. The fit range in each spectral window is set to be around 0.9 cm^{-1} wide, excluding data points at the beginning and end of the laser pulses.

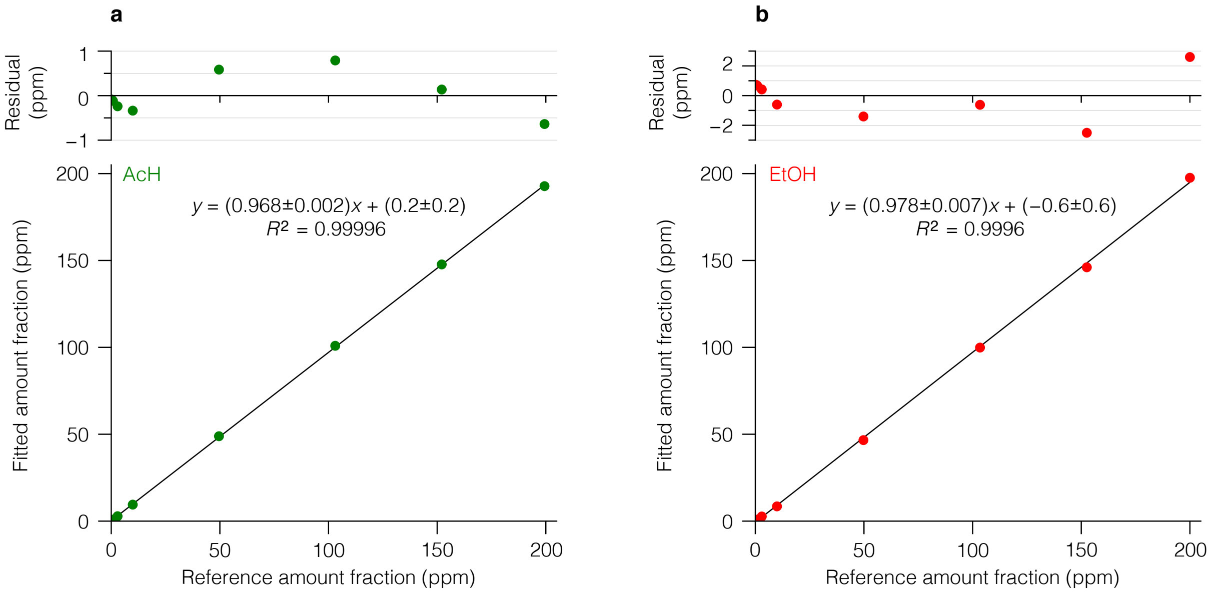

Figure S4: Linearity plots (bottom) for (a) AcH and (b) EtOH along with the corresponding residuals (top). The solid lines show a least-squares linear regression. The error bars (two-sigma deviation) are smaller than the marker size.

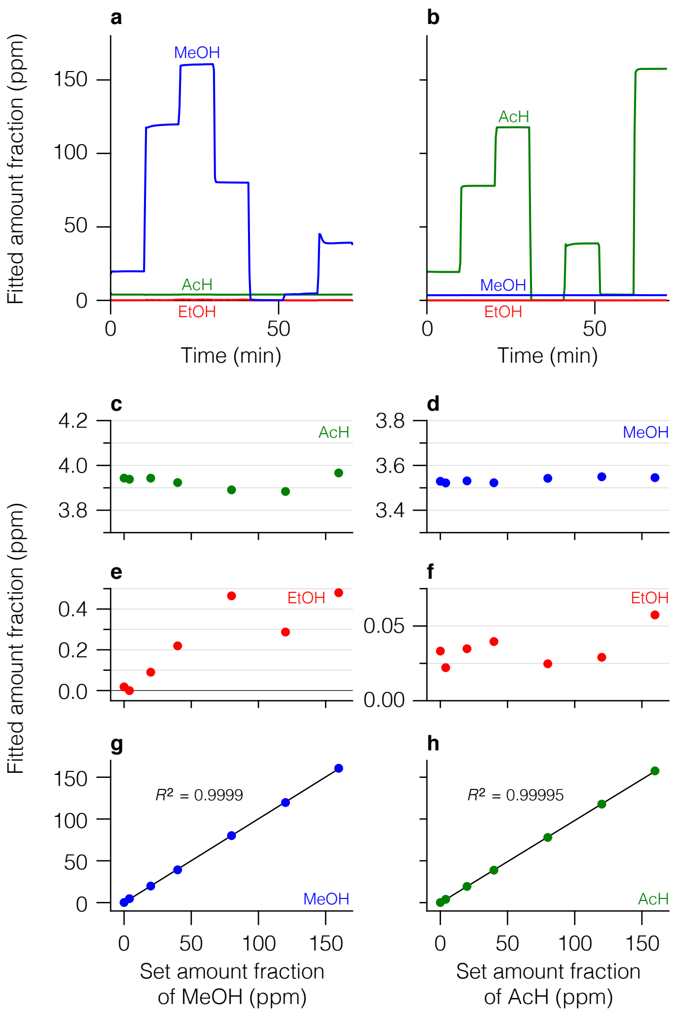

Figure S5: Selectivity study for AcH–MeOH mixtures at amount ratios between 1:40 and 40:1. (a) Time series of fitted amount fractions of the three benchmark compounds when AcH is kept constant at around 4 ppm while the amount fraction of MeOH is varied between 0 and 160 ppm and EtOH is absent. (c,e,g) Fitted amount fractions (colored dots) of the three compounds, averaged over the last 3 min of each step in panel (a) and ordered as a function of the set amount fraction of MeOH. (b) same as (a) but with MeOH and AcH as the constant and the varied compound, respectively. (d,f,h) same as (c,e,g) but extracted from panel (b) and ordered as a function of the set amount fraction of AcH. The solid lines in panels (g,h) represent the fitted linear regression. In panels (b,d) the plotted amount fractions of MeOH were drift-corrected as outlined in Figure S7a.

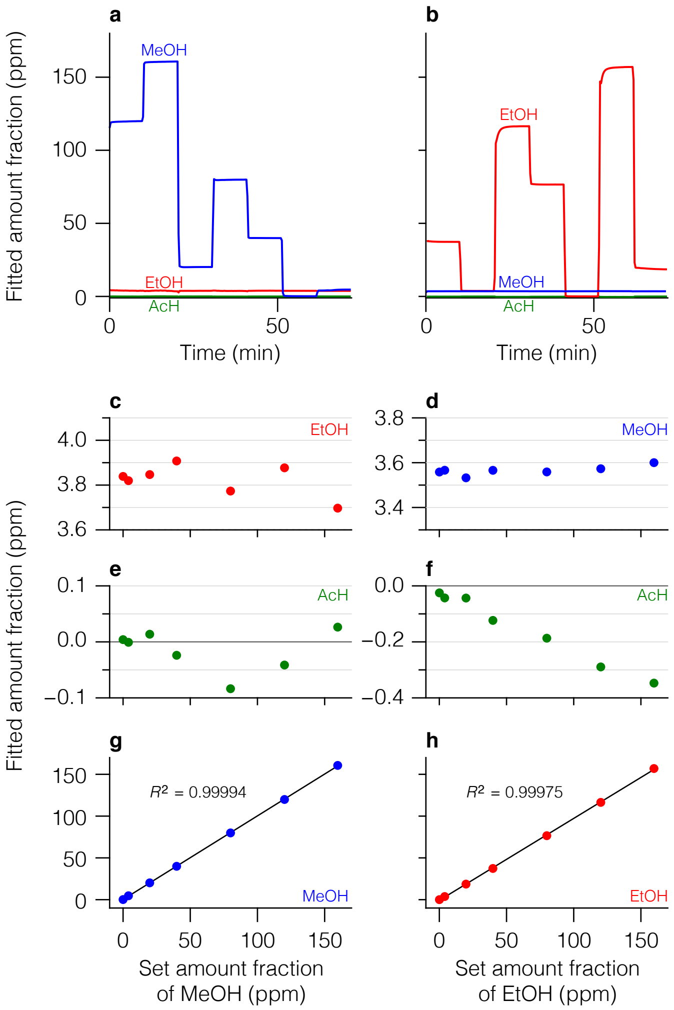

Figure S6: Selectivity study for EtOH–MeOH mixtures at amount ratios between 1:40 and 40:1. (a) Time series of fitted amount fractions of the three benchmark compounds when EtOH is kept constant at around 4 ppm while the amount fraction of MeOH is varied between 0 and 160 ppm and AcH is absent. (c,e,g) Fitted amount fractions (colored dots) of the three compounds, averaged over the last 3 min of each step in panel (a) and ordered as a function of the set amount fraction of MeOH. (b) same as (a) but with MeOH and EtOH as the constant and the varied compound, respectively. (d,f,h) same as (c,e,g) but extracted from panel (b) and ordered as a function of the set amount fraction of EtOH. The solid lines in panels (g,h) represent the fitted linear regression. In panels (b,d) the plotted amount fractions of MeOH were drift-corrected as outlined in Figure S7b.

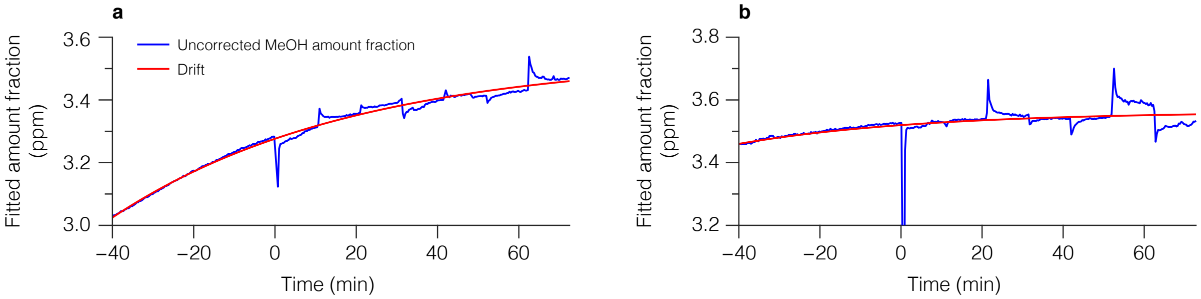

Figure S7: Drift correction applied to the fitted MeOH amount fractions in the selectivity experiments for (a) AcH–MeOH and (b) EtOH–MeOH mixtures when MeOH was the constant compound. The fitted amount fraction of MeOH (blue lines) was monotonously increasing during a purging routine with 4 ppm of MeOH (time between −90 min and 0 min) and in the following selectivity experiments with the same constant amount fraction of MeOH (time between 0 min and 70 min). Such drifts are attributed to adsorption in our gas-delivery system and were generally more pronounced for MeOH than for AcH and EtOH. To evaluate the selectivity of the spectroscopic instrument despite these superimposed drifts, we fitted an exponential equilibration function (red lines) of the form $y(t) = y_\infty - a\exp(-bt)$ with fitting parameters y_∞, a, and b to the uncorrected MeOH amount fraction over the entire plot range. In Figure S5b,d and Figure S5b,d, the fitted MeOH amount fraction is then corrected by addition of $a\exp(-bt)$. For all other results shown throughout this work, no drift corrections were necessary.
